# Distributed lag inspired machine learning for predicting vaccine-induced changes in COVID-19 hospitalization and intensive care unit admission

**DOI:** 10.1038/s41598-022-21969-9

**Published:** 2022-11-05

**Authors:** Atikur R. Khan, Khandaker Tabin Hasan, Sumaiya Abedin, Saleheen Khan

**Affiliations:** 1 Department of Management, North South University, Dhaka, 1229 Bangladesh; 2 Department of Computer Science, American International University of Bangladesh, Dhaka, 1229 Bangladesh; 3 Department of Population Science, University of Rajshahi, Rajshahi, 6205 Bangladesh; 4 Department of Economics, Minnesota State University, Mankato, MN 56001 USA

**Keywords:** Epidemiology, Epidemiology

## Abstract

Distributed lags play important roles in explaining the short-run dynamic and long-run cumulative effects of features on a response variable. Unlike the usual lag length selection, important lags with significant weights are selected in a distributed lag model (DLM). Inspired by the importance of distributed lags, this research focuses on the construction of distributed lag inspired machine learning (DLIML) for predicting vaccine-induced changes in COVID-19 hospitalization and intensive care unit (ICU) admission rates. Importance of a lagged feature in DLM is examined by hypothesis testing and a subset of important features are selected by evaluating an information criterion. Akin to the DLM, we demonstrate the selection of distributed lags in machine learning by evaluating importance scores and objective functions. Finally, we apply the DLIML with supervised learning for forecasting daily changes in COVID-19 hospitalization and ICU admission rates in United Kingdom (UK) and United States of America (USA). A sharp decline in hospitalization and ICU admission rates are observed when around 40% people are vaccinated. For one percent more vaccination, daily changes in hospitalization and ICU admission rates are expected to reduce by 4.05 and 0.74 per million after 14 days in UK, and 5.98 and 1.04 per million after 20 days in USA, respectively. Long-run cumulative effects in the DLM demonstrate that the daily changes in hospitalization and ICU admission rates are expected to jitter around the zero line in a long-run. Application of the DLIML selects fewer lagged features but provides qualitatively better forecasting outcome for data-driven healthcare service planning.

## Introduction

Distributed lags regulate the characteristics of a time series and a DLM is used to infer the short-and long-run dynamic behavior between the predictor and response variables^1–3^. Regression based inflexible statistical learning methods like DLM are used to conduct statistical inferences. On the other hand, flexible and supervised learning methods such as regression tree (RT), random forest (RF), support vector regression (SVR), and deep neural network (DNN) are known for their predictive performances^4–7^. In the presence of time-lagged relationship, selection of lag length is one of the key steps in time series modelling. In fact, a well-defined lag length is selected and all lags up to that time period are included in the model. However, this type of selection may not be appropriate in some cases, for example, to explore the dynamic relationship between vaccination and hospitalization rates. Vaccine requires enough time (few days to few weeks) to prompt the immune system to fight against the virus^8–10^. Thus not all lags are deemed to be important in predicting the hospitalization rates in response to vaccination rates, and a DLM with lag selection is preferred in this case. Akin to the DLM, distributed lags are likely to affect the machine learning and our main objective in this paper is to explore the distributed lag inspired machine learning (DLIML) in forecasting COVID-19 hospitalization and ICU admission rates.

A time series of length *n* may form $n-1$ lagged features to express the *n*th term of the response as a function of the $n-1$ lagged features. If all the lagged features $\{ x_{n-k}: k=1, \ldots , n-1 \}$ are considered in predicting the *n*th term $x_n$, the number of features simply exceeds the effective sample size. In practice, dimensionality problem arises for a univariate time series when the number of it’s lagged features exceeds the half of the series length for decomposition of trajectory (lagged feature) matrix^11^. If the data generating mechanism is unknown in real world scenarios, we may apply dimension reduction method for feature engineering^12,13^ to enhance the predictive performance of any supervised learning algorithm. Since the number of lagged features and effective sample size are inversely related, passing a higher number of lagged features through any dimension reduction technique will reduce the effective sample size. Alternatively, we may reduce the search space for lagged features by evaluating distributed lags in the DLM to explore any short-run dynamic and long-run cumulative effects.

Though a search for distributed lags may be completely data-driven, some background knowledge may provide an insight regarding the search space. Some earlier studies have shown that hospitalization and ICU admission rates are affected by the widespread vaccination for infectious diseases like influenza, human papilloma virus infection, and COVID-19 infection^9,14,15^. A negative association has been found between the vaccination rate and hospitalization rate in different states in USA^16^. Compared to unvaccinated individuals, significantly lower hospitalization and ICU admission rates are found for vaccinated individuals in Bahrain^17^. Though COVID-19 vaccination has been found to decline the hospitalization and ICU admission numbers, a time delay of around two weeks has been found in some studies to observe these effects^8,9^. Thus the observational studies in hospitals reveal a time-lagged relationship of vaccination and its impact on hospitalization. Given the nature of time-lagged relationship, not all lagged features will play important roles in predicting the hospitalization and ICU admission rates. Thus a vaccine-induced DLM and DLIML will be utilized to explore the short-and long-run effects of vaccination on hospitalization and ICU admission rates, and we utilize these relationships further to forecast daily changes in hospitalization and ICU admission rates in UK and USA.

## Data and methods

**Figure 1:**
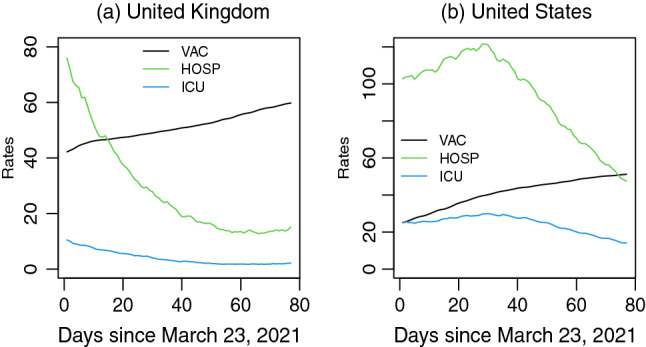
Percentage of people received at least one dose of vaccine (vaccinated), daily death per million (death), patients in hospitals per million (hospitalized), and patients in intensive care unit per million (ICU) since March 23, 2021.

We have extracted vaccination data along with the daily number of admissions in hospital and ICU on June 15, 2021 from the publicly available website https://ourworldindata.org/covid-vaccinations discussed in^18^. After data cleaning, we only obtain enough data on vaccination, hospitalization, and ICU admissions for UK and USA. In our study, we have considered daily time series of length $n=77$ days (11 weeks) from March 23, 2021. Three time series that we have examined to explore the time-lagged relationships and dynamics of daily changes are: the percentage of population received at least one dose of COVID-19 vaccine (*VAC*), the number of patients in hospital per million (*HOSP*), and the number of ICU admissions per million (*ICU*). These time series shown in Fig. 1 demonstrate that as the vaccination rate increases, the hospitalization and ICU admission rates decrease over time.

In the next subsequent sections, we explore dynamic relationships among these three time series both for dynamic marginal and long-run cumulative effects in response to changes in vaccination rates. Later, we utilize the dynamic relationships for forecasting the growths of hospitalization and ICU admission rates. Thus we split the data into training and test time series with the first 10 weeks (70 days) data as training data for model building and the last week’s data as test data for model evaluation.

## Distributed lags for vaccination rates

Vaccination is one of the most preferred options to reduce the transmission and control an epidemic. Vaccine produces antibody in the body that fights against the virus and prompts immune system to respond to a pathogen. A vaccinated person’s immune system becomes more ready to fight against a pathogen and is less likely to suffer from serious illness even if exposed to the pathogen. Though it was thought to be an illusionary assumption at the beginning without enough data on COVID-19 vaccine-induced population level immunity to reduce the transmission to return back to normalcy towards pre-COVID-19 state, it was hypothesized to reduce the severity of the disease^19^. Akin to other infectious diseases such as influenza infection^14^, mass vaccination would likely to reduce the number of critically infected patients requiring hospital admission or ICU support.

Once the first dose of vaccine is received, body starts producing antibody and it takes some times to prompt immune system to respond to a pathogen. In a study on antibody response to seronegative and seropositive persons from single dose *mRNA* vaccine, seronegative persons are found to have relatively low *SARS-CoV-2 IgG* responses within 9–12 days after vaccination whereas seropositive persons are found to develop high antibody titers within days even in some cases within 4 days. However, higher degrees of response to single dose vaccine is reported for seronegative persons around a time lag of 20 days^20^. In an early study in USA, all participants were found to develop detectable SARS-CoV-2 IgG antibodies in serum samples by 15 days following the first vaccination dose^21^. These results demonstrate that there would be a time-lagged relationship between vaccination and hospital admission rates. As more and more people become vaccinated over time, fewer number of patients would require hospital admission or ICU support. Thus a dynamic model can be used to explore the time-lagged relationships of hospitalization and ICU admission rates with vaccination rates.

A DLM that explores the effect of a regressor *x* on *y* over time can be expressed as

1 $$\begin{aligned} y_t = \alpha + \sum _{s=0}^{q} \beta _s x_{t-s} + \epsilon _t, \end{aligned}$$

where $\epsilon _t$ is a stationary term with $E(\epsilon _t) = 0$, $Var(\epsilon _t) = \sigma ^2$, and $Cov(\epsilon _t, \epsilon _s) =0$ for $t \ne s$. The lag weights $\beta _s$ for $s=1, \ldots , q$ collectively represent the lag distribution and define the pattern how *x* affects *y* over time^1,2,22^. The dynamic marginal effect of *x* on *y* at the *s*th lag is

2 $$\begin{aligned} \frac{\delta y_{t+s}}{\delta x_t} = \frac{\delta y_{t}}{\delta x_{t-s}} = \beta _s, \end{aligned}$$

for $s=1, \ldots , q$. The dynamic marginal effect is essentially an effect of temporary change in *x* on *y*, whereas the long-run cumulative effect $\sum _{s=1}^{q}\beta _s$ measures how much *y* will be changed in response to a permanent change in *x* when both *x* and *y* are stationary^1^.

Assuming $x_t$ is the vaccination rate and $y_t$ is the hospitalization rate in Eqs. (1)–(2), we may explore the temporary dynamic marginal effect and long-run cumulative effect of vaccination on hospitalization rates. Similarly, we may compute the temporary dynamic marginal effect and long-run cumulative effect of vaccination on ICU admission rates.

### Daily changes in hospitalization per million

The daily change in hospitalization per million is $\triangle HOSP_t = HOSP_{t} - HOSP_{t-1}$ and the daily change in vaccination rate is $\triangle VAC_t = VAC_{t} - VAC_{t-1}$, where $t=2, \ldots , n$. Akin to the DLM in Eq. (1), we may define a dynamic model as

3 $$\begin{aligned} \triangle HOSP_{t} = \alpha + \sum _{s \in I} \beta _s \triangle VAC_{t-s} + \epsilon _t, \end{aligned}$$

where $s \in I$ refers to a lag distribution that consists of time lags from the set of integers and not all $\beta _s$ contribute significantly as the vaccine requires some times to prompt the immune system to respond to the pathogen. Estimates of parameters for a DLM of $\triangle HOSP$ are provided in Table 1.

**Table 1:** Distributed lag model for hospitalization ($\triangle HOSP$) in UK and USA. Here, the intercept term refers to $\alpha$ in Eq. (3).

Country	Model term	Estimate	*p*-value
UK	(Intercept)	− 1.5013	0.0000
	$\triangle VAC_{t-4}$	2.0530	0.0204
	$\triangle VAC_{t-9}$	4.5311	0.0000
	$\triangle VAC_{t-13}$	2.5950	0.0457
	$\triangle VAC_{t-14}$	− 4.0476	0.0018
USA	(Intercept)	− 2.2786	0.0000
	$\triangle VAC_{t-4}$	5.4082	0.0000
	$\triangle VAC_{t-16}$	4.7100	0.0013
	$\triangle VAC_{t-17}$	− 4.7050	0.0288
	$\triangle VAC_{t-18}$	4.4492	0.0101
	$\triangle VAC_{t-20}$	− 5.9836	0.0000

**Figure 2:**
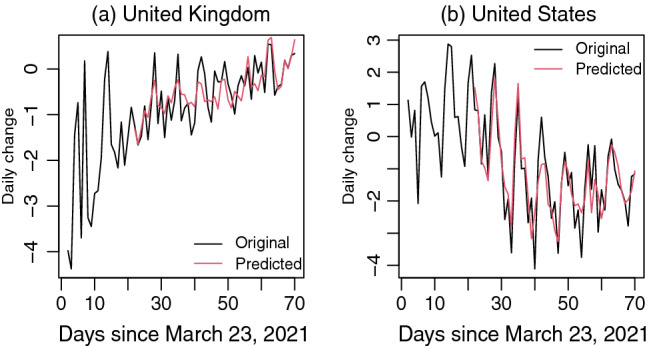
Original and predicted $\triangle HOSP$ since March 23, 2021 in (**a**) United Kingdom and (**b**) United States.

Vaccination is supposed to reduce the hospital admission rates. We have found that a temporary dynamic marginal effect is negative only around or after the 14th lag in the DLM of $\triangle HOSP$ for UK and USA. A positive dynamic marginal effect refers to the increase of $\triangle HOSP$ whereas a negative dynamic marginal effect refers to the decrease of $\triangle HOSP$ in response to lagging $\triangle VAC$. For one percent increase in the daily vaccination (one unit increase in $\triangle VAC$) in UK, daily change in hospitalization rate ($\triangle HOSP$) is decreased by 4.05 after the 14th day. The dynamic temporary marginal effects on $\triangle HOSP$ and $\triangle ICU$ in USA become negative after the 17th day and 20th day, respectively. This is an indication of 4.70 per million reduction in $\triangle HOSP$ for one percent increase in the daily vaccination (one unit increase in $\triangle VAC$) in USA in 17 days apart. Similarly, a one percent increase in vaccination rate in USA seems to result in 1.04 per million reduction in $\triangle ICU$ after the 20th day. Figure 2 shows that the original and predicted $\triangle HOSP$ are mostly negative with some fluctuations around zero over time, which provides an insight that a positive dynamic marginal effect results in less negative changes whereas a negative dynamic marginal effect results in more negative changes in hospitalization rates. Regardless of more or less negative changes in hospitalization rates in response to dynamic marginal effect of changes in vaccination rates, long-run cumulative effects ($\sum \hat{\beta }_s = 5.1315$ for UK and $\sum \hat{\beta }_s = 3.8788$ for USA) will yield less negative change in hospitalization rates over time. Thus the vaccination is going to significantly reduce the hospitalization rates in long-run. As more and more people are vaccinated, $\triangle VAC$ will tend to zero over time and $\triangle HOSP$ is expected to jitter around the zero line in a long-run.

### Daily changes in ICU admission per million

Akin to the DLM in Eq. (1), we may define a dynamic model for ICU admission rates as

4 $$\begin{aligned} \triangle ICU_{t} = \alpha + \sum _{s \in I} \beta _s \triangle VAC_{t-s} + \epsilon _t, \end{aligned}$$

where $s \in I$ forms a lag distribution as has been explained in Eq. (3), and $\triangle ICU_t = ICU_t - ICU_{t-1}$ for $t=2, \ldots , n$. Estimates of parameters from this model are shown in Table 2.

**Table 2:** Distributed lag model for ICU admission ($\triangle ICU$) in UK and USA. Here, the intercept term refers to $\alpha$ in Eq. (4).

Country	Model term	Estimate	*p*-value
UK	(Intercept)	− 0.1106	0.0336
	$\triangle VAC_{t-13}$	0.5654	0.0755
	$\triangle VAC_{t-14}$	− 0.7366	0.0288
	$\triangle VAC_{t-16}$	0.9321	0.0005
	$\triangle VAC_{t-19}$	− 0.5457	0.0006
USA	(Intercept)	− 0.5858	0.0000
	$\triangle VAC_{t-16}$	1.1634	0.0000
	$\triangle VAC_{t-18}$	0.8003	0.0027
	$\triangle VAC_{t-20}$	− 1.0427	0.0000

**Figure 3:**
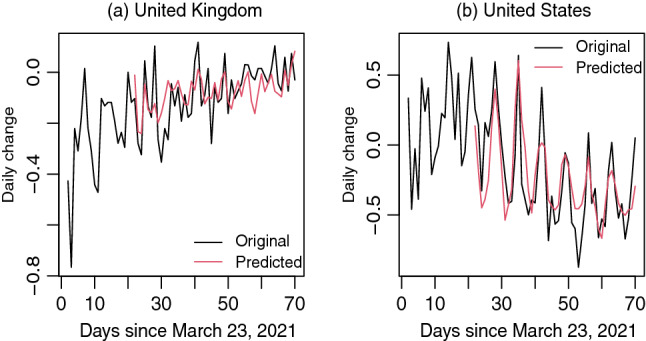
Original and predicted changes in ICU admission ($\triangle ICU$) since March 23, 2021 in (**a**) United Kingdom and (**b**) United States.

A minimum of 2 weeks (14 days) lag is found significant in the DLM of $\triangle ICU$ in response to $\triangle VAC$. Thus any daily change in ICU admission can be explained by the changes in vaccination rates with a dynamic marginal effects of 2 weeks or more time lags. As can be seen in Fig. 3, daily changes in ICU admission rates per million are mostly negative with some jittering changes around zero over time. Thus any positive dynamic marginal effect will yield less negative change and any negative dynamic marginal effect will incur more negative change in ICU admission rates. The long-run cumulative effect (sum of dynamic marginal effects, $\sum \hat{\beta }_s = 0.2152$) of $\triangle VAC$ on $\triangle ICU$ for UK is positive, which mimics that the daily changes in ICU admission ($\triangle ICU$ ) will be less negative over time. The long-run cumulative effect ($\sum \hat{\beta }_s = 0.9210$) of $\triangle VAC$ on $\triangle ICU$ for US is also positive. Thus, in a long-run, changes in ICU admission ($\triangle ICU$ ) will be jittering around zero as more and more people become vaccinated rendering the daily changes in vaccination rates ($\triangle VAC$) to zero.

## Sliding window correlation

Dynamic relationships between two time series may lead to a functional connectivity where the connectivity may exhibit dynamic changes within a time scale^23,24^. We already have explored the dynamic relationship between vaccination rates and hospitalization rates where the functional relationship has been expressed by the DLM. However, the functional connectivity changes over time as the dynamic marginal effects can be positive or negative. Such changes in dynamic relationship between two time series can be examined by computing correlation between two lagged variables over a sliding window^23,24^.

**Figure 4:**
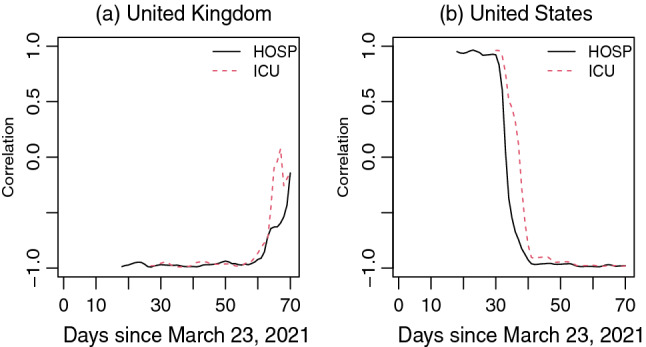
Dynamic correlation of hospitalization per million and ICU patients per million with lagged vaccination rates in (**a**) United Kingdom and (**b**) United States.

As the sliding window crawls over the series of length *n* with a window size *m* and time lag *k*, we can compute $n-(m+k)+1$ correlations corresponding to the time points $(m+k), \ldots , n$. These correlations show the time dependent changes in dynamic relationship between two time series. Since the DLM of $\triangle HOSP$ on $\triangle VAC$ provides coefficients corresponding to the significant time lag and the smallest lag in the model is $s=4$, we choose $k=4$ and $m=14$ (2 weeks window) to obtain sliding window correlation to explore the dynamic relationship between *HOSP* and *VAC*. Similarly, we compute sliding window correlation between *ICU* and *VAC* time series. Computed sliding window correlations are shown in Fig. 4.

By comparing the correlation curves in Fig. 4 for UK and USA, we find that the correlations are almost same around the 50*th* days. Though the correlation curves in US show highly positive correlations at the beginning, both curves show sharp decline with highly negative correlation around the 40*th* day. Such dynamic nature in correlation can be characterized with the fewer number of vaccinated people at the beginning that could not cause significant reduction in hospitalization and ICU admission rates. As the time passes, more people become vaccinated and highly negative correlation reflects huge reduction in COVID-19 patients requiring hospitalization and ICU admission. More than 40% people become vaccinated (received at least one dose of vaccine for COVID-19) by the $50-(m+k) = 32$th day in USA and a sharp decline of correlation is achieved afterwards. On the other hand, correlation curves seem to be very close to − 1 at the beginning and keeps rising further after the 60*th* day in UK. This dynamic nature of correlation can be an effect of high vaccination rate in UK at the beginning of this study period. Because of a fast track vaccination, more than 40% people become vaccinated in UK soon after the vaccination campaign and highly negative correlations are observed both for the hospitalization and ICU admission rates even from the beginning of our study period. However, hospitalization and ICU admission rates do not decrease too much once more than 50% people are vaccinated, which results in more deviation of correlation from − 1.

## Distributed lag inspired machine learning

We already have explored that not all lagged features are significant in the fitted DLMs. Consequently, consecutive lag orders may or may not be found significant in DLMs. As has been shown in Table 1, DLM of $\triangle HOSP_t$ for the predictor $\triangle VAC_{t-k}$ has a lag distribution of $k \in \{ 4,9,13,14 \}$ for UK data, whereas the DLM for USA data has a lag distribution of $k \in \{ 4,16,17,18,20 \}$. Such distributions are observed because of the exclusion of many redundant lagged features from these models. Moreover, almost 75% of lagged features are found redundant in the DLMs in Tables 1 and 2. Thus we explore distributed lags in machine learning for prediction of $\triangle HOSP$ and $\triangle ICU$.

### Distributed lags in regression tree

A regression tree is built through a process of binary recursive partitioning. Input variables are recursively partitioned by values until the terminal nodes and prediction of response variable is made by estimating a regression function. However, inclusion of irrelevant input variables (features) may affect the predictive performance of the model by increasing the mean square error (MSE). So, variable (feature) importance score is computed by evaluating the reduction of MSE attributed to each feature at each split. A higher importance score refers to more relevance of the feature in predicting the response variable^25,26^.

**Figure 5:**
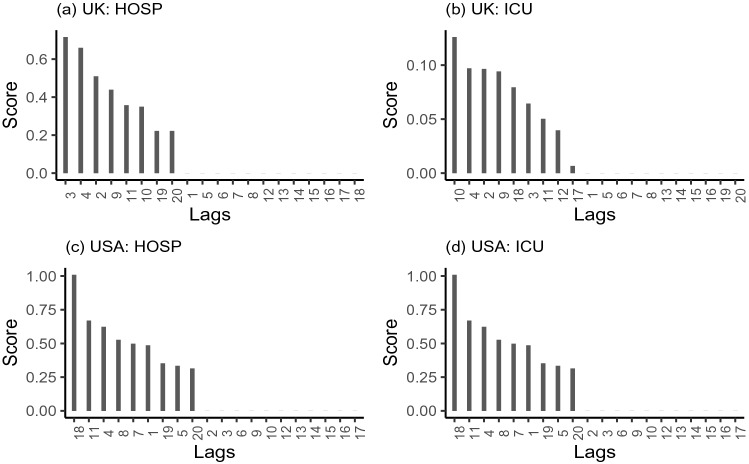
Average feature importance scores computed from 500 replications of RT model of $\Delta HOSP_t$ and $\Delta ICU_t$ for distributed lags of $\Delta VAC_{t-k}$ in USA and UK, where $k=1,\ldots ,20$.

Importance scores of lagged features in RT models are shown in Fig. 5. Not all lagged features are found to contribute in improving the model. The distributed lags $k \in \{ 3,4,2,9,11,10,19,20\}$ of $\triangle VAC_{t-k}$ are found to be important for predicting $\triangle HOSP_t$ and $k \in \{ 10,4,2,9, 18, 3, 11, 12, 17 \}$ are found to be important for predicting $\triangle ICU_t$ from UK data. Similarly, when RT is implemented to USA data, the distributed lags $k \in \{ 18, 11, 4, 8, 7, 1, 19, 5, 20 \}$ and $k \in \{ 8, 1, 7, 14, 19, 11, 4, 12, 10, 18 \}$ are found to be important for predicting $\triangle HOSP_t$, and $\triangle ICU_t$ respectively in response to $\triangle VAC_{t-k}$.

### Distributed lags in random forest

**Figure 6:**
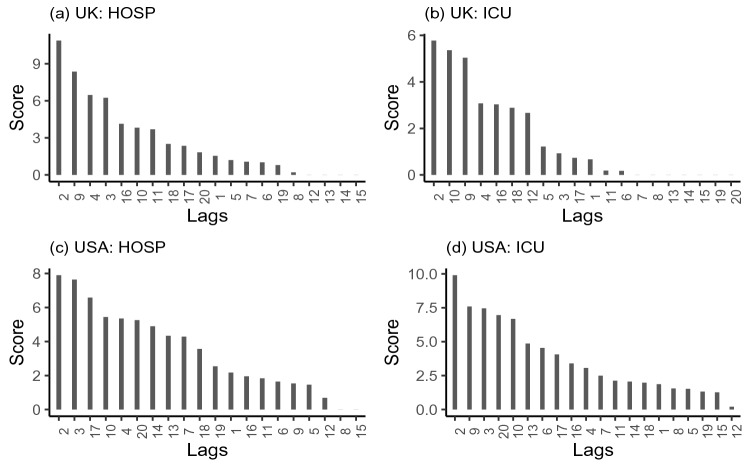
Average feature importance scores computed from 500 replications of RF model of $\Delta HOSP_t$ and $\Delta ICU_t$ for distributed lags of $\Delta VAC_{t-k}$ in USA and UK, where $k=1,\ldots ,20$.

For regression with a RF, the MSE is computed on the out-of-bag data for each tree, and then the same is computed after permuting a variable. The differences are averaged and normalized by the standard error to compute an overall importance score. By randomly permuting a feature, original association with the response is broken and the inclusion of permuted feature in the RF model with other non-permuted features increases the MSE. Thus a feature with higher level of importance score is deemed to have a higher level of contribution in predicting the response variable^27^. Computed feature importance score from RF are provided in Fig. 6.

Unlike the selection of fewer distributed lags in RT and DLM, implementation of RF identifies more lagged features in Fig. 6 for prediction of response variables. It seems that the importance scores are tailing off slowly in Fig. 6 and we are to consider many features as predictors. Distributed lags of $\{ 2, 9, 4, 3, 16, 10, 11, 18, 17, 20, 1, 5, 7, 6, 19, 8 \}$ and $\{2, 10, 9, 4, 16, 18, 12, 5, 3, 17, 1, 11, 6 \}$ are found to be important for the prediction of $\triangle HOSP$ and $\triangle ICU$ in UK, respectively. Though lag distribution of $\{2, 3, 17, 10, 4, 20, 14, 13, 7, 18, 19, 1, 16, 11, 6, 9, 5, 12 \}$ is deemed to be important for $\triangle HOSP$, a lag distribution consisting all 20 lags under study are preferred for the prediction of $\triangle ICU$ in USA.

### Distributed lags in support vector regression

Effect of lag distributions (subsets of lagged features) in SVR can be evaluated by using the recursive feature elimination (RFE) procedure^25,28^. The RFE eliminates features recursively from the full model and selects a subset of the most important features. At each stage of the search, the least important features are eliminated prior to rebuilding the model with the remaining features. Models are evaluated at each iteration until the best subset of feature is selected by using an appropriate objective function^29^. The best subset is the one that produces the least root mean squared error (RMSE).

**Figure 7:**
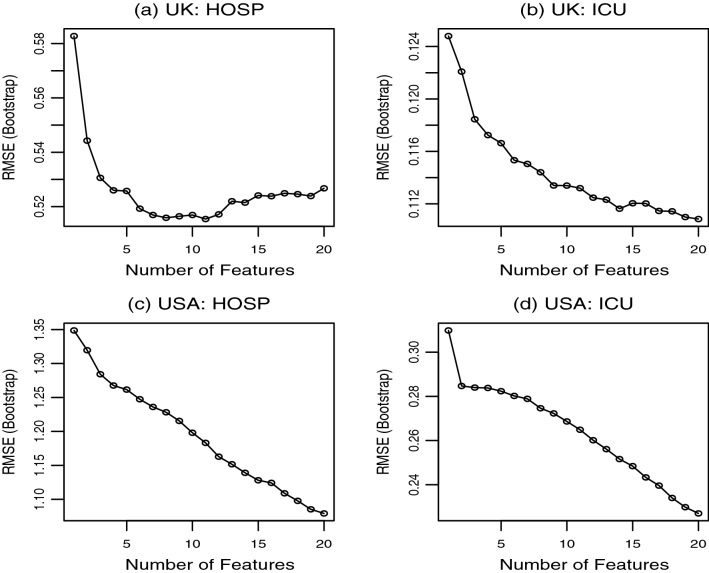
Number of features selected by recursive feature elimination in SVR model of $\Delta HOSP_t$ and $\Delta ICU_t$ for distributed lags of $\Delta VAC_{t-k}$ in USA and UK, where $k=1,\ldots ,20$. Distributed lags $\{2, 3, 4, 9, 10, 11, 17, 18\}$ are selected for (**a**), and $\{1,2, \ldots , 20 \}$ are required for figures in (**b**)–(**d**).

Figure 7 shows RMSE computed as an average across 500 replications (runs) of SVR model. A subset of 8 lagged features with distributed lags $\{ 2, 3, 4, 9, 10, 11, 17, 18 \}$ is found to provide the least RMSE whilst predicting $\triangle HOSP$ in UK. For each of the remaining responses $\triangle ICU$ in UK, and $\triangle HOSP$ and $\triangle ICU$ in USA, the least RMSFE is achieved when all 20 lagged features are considered.

### Distributed lags in deep neural networks

**Figure 8:**
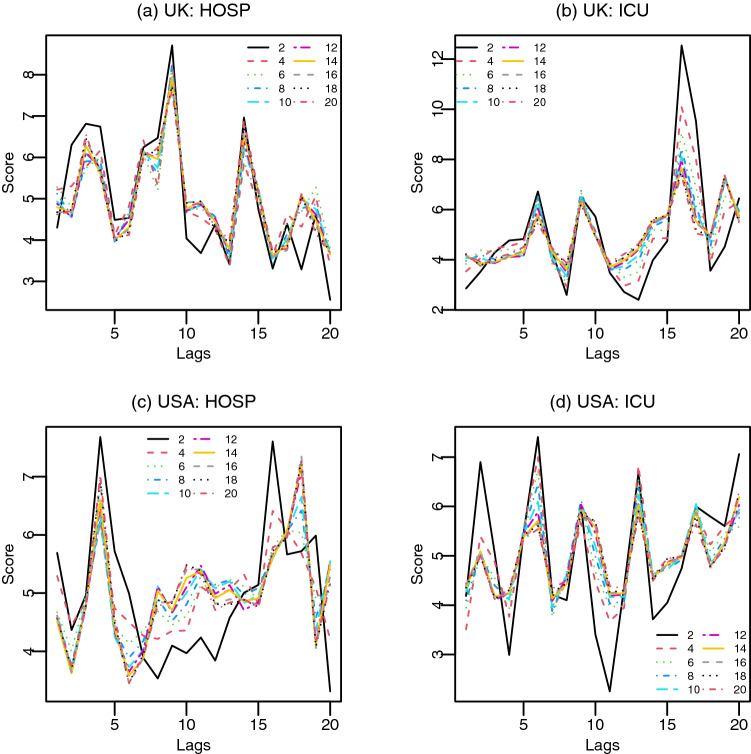
Average feature importance scores computed from 500 replications of NN model with single hidden layer to predict $\Delta HOSP_t$ and $\Delta ICU_t$ by using $\Delta VAC_{t-k}$ in USA and UK, where $k=1,\ldots ,20$. Lines show average scores of lagged features for different number of units ($2, 4, \ldots , 20$) in hidden layers.

By construction, neural network (NN) assigns lower weights to features having lower discriminating power (lower contribution in prediction) during the generation of non-linear combinations of features for prediction. Within a search space of 20 lagged features, neural networks are likely to have enough information to learn the features without making many (or any) of these features redundant. More importantly, number of units and hidden layers play important roles in assigning weights to features. For instance, we consider a single hidden layer with different number of units in NN to explore the importance of these lagged features^25,30^. Results shown in Fig. 8 demonstrate the effects of number units in a single hidden layer. As the number of units in a hidden layer changes, the importance scores of features also change. As the number of hidden layers increases, effects of layers and number of units in hidden layers become more stringent, because a feature from one layer is passed to the next layer. Thus we may consider all 20 lagged features with distributed lags $k \in \{1, 2, \ldots , 20 \}$ of $\triangle VAC_{t-k}$ for the prediction of $\triangle HOSP_t$ and $\triangle ICU_t$, where $t=1, 2,\ldots , n$.

## Forecasting future changes in hospitalization and ICU admission rates

It is well recognized that inflexible learning methods such as regression models (DLMs) are preferable for statistical inferences to examine the significance of dynamic marginal effects and flexible learning methods such as RT, RF, SVR, and DNN are preferable for prediction of respiratory tract infection (RTI) and COVID-19 time series^5,6,31,32^. DLM based inferences have explored that not all lagged features derived from $\triangle VAC$ affect the $\triangle HOSP$ and $\triangle ICU$ significantly. Thus we have explored the distributed lags for machine learning models in the previous sections. These distributed lagged features have been used in machine learning to obtain forecasts for daily changes in hospitalization and ICU admission rates.

We train machine learning models with the training data, select the best model, and apply the selected model to evaluate the forecasting performance based on the out-of-sample data. Forecasts are compared with the corresponding original values and mean squared forecast error (MSFE) is computed for each of the models by using the formula

5 $$\begin{aligned} MSFE = \frac{1}{h} \sum _{i=n+1}^{n+h}\left( y_i - \hat{y}_i \right) ^2, \end{aligned}$$

where *h* is the number of out-of-sample forecast, *n* is current time point, and $\hat{y}_i$ is the forecast corresponding to the original value $y_i$ for *h* future (out-of-sample) time points $i=n+1, \ldots , n+h$. We prefer a model that provides the least MSFE in forecasting^33,34^.

We optimize models under different parameter settings and provide the evaluation results only based on the best tuned models. For example, we have evaluated DNN by computing MSE for different combinations of number of hidden layers, batch sizes, and node sizes of hidden layers. We adopt similar selection procedures for SVR by searching parameters over a grid. Since the variations and dynamic patterns of $\triangle HOSP$ and $\triangle ICU$ time series for UK and USA are different, different models are found to tune-up for different series. Machine learning models also incorporate randomness by design. For example, randomness in neural networks can be incorporated due to the randomness in initialization of weights, regularization, embedding of layers, and stochastic optimization. Similarly, randomness in RF is occurred because of the random partition of data to create forest of regression trees. Because of such randomness, different runs of the same model on the same data produce different predictions. Thus the performance measure MSFE from a single run is not suitable for comparison across a set of models. So, we repeat the execution 500 times, obtain predictions and compute MSFE from each execution, and compute the average MSFE across 500 runs (repeated executions) to compare the predictive performance of different machine learning models. Average MSFE values computed from RT, RF, SVR, and DNN are provided in Table 3.

**Table 3:** MSFE from out-of-sample prediction. Here, DLIML is the distributed lag inspired model and ReMSE is the ratio of MSFE from DLIML and Full models.

Response	Model	UK			USA		
		DLIM	Full	ReMSE	DLIM	Full	ReMSE
$\triangle HOSP$	RT	0.3741	0.3783	0.9889	0.5414	0.6523	0.8300
	RF	0.3118	0.3208	0.9719	0.4626	0.4617	1.0019
	SVR	0.2284	0.3954	0.5776	0.5588	0.5588	1.0000
	DNN	0.5050	0.5050	1.0000	0.0277	0.0277	1.0000
$\triangle ICU$	RT	0.0217	0.0229	0.9476	0.0571	0.0683	0.8360
	RF	0.0118	0.0130	0.9077	0.0258	0.0263	0.9810
	SVR	0.0211	0.0211	1.0000	0.0152	0.0152	1.0000
	DNN	0.0188	0.0188	1.0000	0.0277	0.0277	1.0000

Computed relative mean squared forecast error (ReMSE) shown in Table 3 demonstrate that the DLIML provides qualitatively similar (value close to 1.00) or better outcome (value less than 1.00) when compared with the full model. Though the DLIML has selected a fewer number (or at most equal number of features) of lagged features compared to the full model, it does not compromise the forecasting performance. For the prediction of daily changes in hospitalization ($\triangle HOSP$) and ICU admission ($\triangle ICU$) rates in USA, DNN and SVR models provide the least MSFE when both the DLIML and full models are evaluated. In both of these cases, DLIML demonstrates significant contribution for all lagged features under study and provides MSFE equal to that obtained from the full model. On the other hand, when DLIML is implemented to UK data, SVR is found as the best model for $\triangle HOSP$ with almost 42% reduction in MSFE compared to the full model. Similarly, RF is found to better forecast $\triangle ICU$ in UK where the application of DLIML results in almost 10% reduction in MSFE.

## Conclusion

Vaccination is found to reduce the hospitalization and ICU admission rates for COVID-19 patients. However, this effect is not observed instantly as vaccines require sufficient time to prompt the immune system. So, there exists a time-lagged relationship of hospitalization and ICU admission rates with vaccination rates. Application of DLM has explored the short-run dynamic effects of distributed lags of vaccination rates on the hospitalization and ICU admission rates. Fitted DLM reveals the long-run cumulative effect of vaccination rates with an indication that hospitalization and ICU admission rates are expected to vary around zero in long-run. This is an indication that the COVID-19 pandemic may not dissipate shortly and hospitalization rates may not dissipate in long-run. Inspired by the distributed lags in DLM, we have examined distributed lags in machine learning models and have applied DLIML to obtain a week ahead forecast. We have demonstrated with RT, RF, SVR and DNN models that the DLIML provides relatively better forecasting outcome even with only a subset of lagged features. A healthcare administrator therefore can utilize the DLIML for forecasting and use these forecasts to learn about future hospitalization and ICU admission rates to prepare a service plan.

## Data Availability

The datasets generated and analyzed during the current study are openly available in the Our World in Data repository, https://ourworldindata.org/covid-vaccinations.
